# Activation recovery interval imaging of premature ventricular contraction

**DOI:** 10.1371/journal.pone.0196916

**Published:** 2018-06-15

**Authors:** Ting Yang, Long Yu, Qi Jin, Liqun Wu, Bin He

**Affiliations:** 1 Department of Biomedical Engineering, University of Minnesota, Minneapolis, MN, United States of America; 2 Department of Cardiology, Shanghai Ruijin Hospital, Shanghai, China; 3 Department of Biomedical Engineering, Carnegie Mellon University, Pittsburgh, PA, United States of America; Universiteit Gent, BELGIUM

## Abstract

Dispersion of ventricular repolarization due to abnormal activation contributes to the susceptibility to cardiac arrhythmias. However, the global pattern of repolarization is difficult to assess clinically. Activation recovery interval (ARI) has been used to understand the properties of ventricular repolarization. In this study, we developed an ARI imaging technique to noninvasively reconstruct three-dimensional (3D) ARI maps in 10 premature ventricular contraction (PVC) patients and evaluated the results with the endocardial ARI maps recorded by a clinical navigation system (CARTO). From the analysis results of a total of 100 PVC beats in 10 patients, the average correlation coefficient is 0.86±0.05 and the average relative error is 0.06±0.03. The average localization error is 4.5±2.3 mm between the longest ARI sites in 3D ARI maps and those in CARTO endocardial ARI maps. The present results suggest that ARI imaging could serve as an alternative of evaluating global pattern of ventricular repolarization noninvasively and could assist in the future investigation of the relationship between global repolarization dispersion and the susceptibility to cardiac arrhythmias.

## Introduction

Dispersion of ventricular repolarization due to abnormal activation contributes to the susceptibility to cardiac arrhythmias [1]. It has been shown that calcium plays an important role in cardiac excitation-contraction coupling and calcium leak is determinant in pathological conditions such as heart failure [2]. Studies have shown the relationship between inhomogeneity of ventricular repolarization and enhanced ventricular arrhythmia vulnerability [3–5]. Refractory period, transmembrane action potential duration, and activation recovery interval (ARI) have been used to understand the properties of ventricular repolarization. Compared to the other two measurements, ARI has the advantage of being able to be extracted from multiple, simultaneously recorded electrograms and reflecting the dynamics and spatial characteristics of repolarization.

Wyatt et al. [6] reported to estimate activation and repolarization times from unipolar electrograms. By comparing the difference between the repolarization time and the activation time with simultaneously recorded in vivo transmembrane action potential durations, Wyatt method was shown to be a good estimation of action potential durations (APDs). Later, ARI was clearly defined as the interval between times of activation and repolarization, and was used in two animal studies which demonstrated high correlation between ARI from unipolar electrograms and refractory periods or action potential durations under a variety of conditions [7,8]. In Wyatt method, activation and repolarization time is defined as the time of minimum derivative of the QRS and the time of maximum derivative of the T wave in unipolar electrograms, respectively.

T wave polarities correspond to different repolarized regions, and there has been a controversy over the determination of repolarization time for positive T waves in unipolar electrograms. Chen et al. [9] proposed an alternative method in which the minimum derivative on the downslope of the positive T waves was chosen to represent the local repolarization. This study and other following studies [10,11] have observed a better correlation between ARI and MAP90% (90% repolarization of monophasic action potential recordings). However, the theoretical rationale behind this alternative method remains unclear. To justify the use of Wyatt method, Coronel et al. [12] have presented experimental evidences and Potse et al. [13] have validated through a simple theoretical model that the maximum derivative of T waves should always be used regardless of T wave polarities. And finally, a more recent study [14] identified the sources of measurement bias of Wyatt method and supported previous assertions that alternative method does not reliably represent either repolarization time or MAP90%. So, the determination method of repolarization time in unipolar electrograms from CARTO system to evaluate the performance of ARI imaging in this study is Wyatt method.

Besides validating the use of ARI from invasive recordings, it is desirable to discover parameters that could reflect repolarization properties from noninvasive recordings. It has been shown that indices derived from 12-lead ECG are inadequate [15,16] and even high-resolution body surface potential map (BSPM) has its limit in identifying local changes in repolarization [17]. In this case, cardiac electrical imaging technique comes as a better choice in the sense of being noninvasive and time-saving and providing detailed cardiac activities. Based on the relationship between epicardial potential and body surface potential distribution [18], epicardial potential imaging such as noninvasive electrocardiographic imaging (ECGI) was developed [19] to reconstruct epicardial potentials from BSPMs and has been applied to a variety of conditions [20]. Regularization techniques on solving the epicardial potential have been proposed and compared among the existing techniques [21,22]. Activation time imaging on both the epicardial and endocardial surface was also proposed [23,24] and have been successfully applied to human subjects [25,26]. Most recently, a quantitative comparison was made between ECGI and invasive epicardial electrography in four normal anesthetized dogs for the purpose of reconstructing epicardial activation and recovery patterns [27]. However, since ventricular repolarization is inhomogeneous [28] and one can only infer deep source activities from epicardial potential maps [29], a 3-dimensional (3D) estimation of activation and repolarization would be highly desirable.

In 3D space, the moving dipole model was first used to represent the cardiac activity [30], and it was applied to localize the origins of ventricular activation [31]. Later, distributed source models were proposed and have gained much attention since cardiac electrical activity is distributed throughout the heart. A 3D cardiac electrical imaging (3DCEI) technique [32–35] was developed to estimate the equivalent current density at each cardiac source location from BSPMs and derived the activation time from the time course of current density. This method has been quantitatively evaluated in animal models under different conditions such as pacing, ventricular tachycardia, drug-induced QT prolongation and non-ischemic heart failure [36–41]. 3DCEI was also applied to atrial arrhythmias by extracting frequency features from the reconstructed current density [42]. Several other efforts were made to reconstruct transmembrane potentials in 3D space noninvasively [43–48]. These methods have been tested under cardiac diseased conditions, such as heart failure and myocardial infarction [49–51]. Most recently, a machine learning algorithm was incorporated to localize the onset activation location in a premature ventricular contraction (PVC) patient and five cardiac resynchronization therapy (CRT) patients [52].

Premature ventricular contraction (PVC) is a type of ectopic beat caused by an ectopic pacemaker in the ventricles. It is one of the common arrhythmias and its prevalence varies depending on many factors, such as age, sex, race, and the presence of heart disease. In a large cross-sectional analysis of the 15,792 individuals (aged 45–65 years) from the U.S., the prevalence of PVC is >6% among these adults based on a 2-minute ECG [53]. Pacing is one way to study the cardiac activities during ectopic beats. Studies have shown that ventricular repolarization potential distributions during ectopic beats were influenced by transventricular gradients (gradients from one side of the heart to the other) [54] and the longest ARIs were located at the sites of earliest activation and shortest at the latest activation areas during pacing [10].

3DCEI technique has been extensively validated under a variety of conditions. In this study, we investigated, for the first time, the recovery properties of ventricles by reconstructing ARI distributions from the time course of current density and evaluated 3D ARI imaging technique by comparing the imaged ARI maps with those extracted from CARTO systems.

## Methods

### Patients

BSPMs were recorded in 10 PVC patients (Male = 4, Female = 6, average age = 47.8 ± 11.7 years old) before the electrophysiology (EP) procedure with spontaneous PVCs detected. None of them has undergone ablation procedure before. Patient characteristics and selective medical records are summarized in **Table 1**. The number of PVCs was counted by Holter monitors. Study protocols were approved by the Institutional Review Board of the University of Minnesota and the Shanghai Ruijin Hospital (affiliated with Shanghai Jiao Tong University School of Medicine, Shanghai, China), and all methods were performed in accordance with the relevant guidelines and regulations. All patients provided informed consent to participate in the study.

**Table 1:** Statistics of patient characteristics.

Pt	Age	Gender	HR (bpm)	BP (mmHg)	Antiarrhythmic Medication	Medical History	PVCs/24 h
1	41	F	74	116/77	None	Diabetes	15160
2	53	M	70	105/63	Bisoprolol	Allergic to penicillin	25630
3	43	M	75	121/64	Amiodarone	Hypertentsion	N/A
4	71	F	70	117/61	Aspirin, Lipitor	Atherosclerosis of coronary artery and carotid artery, cervical disc herniation	21100
5	54	F	80	118/67	Moracizine	Allergic to cephalosporin	13441
6	37	M	95	137/86	None	Sublingual gland excision	27161 /23 h
7	42	F	78	115/75	None	None	26337
8	43	M	82	150/80	None	None	39476
9	33	F	84	100/55	None	Cardiac arrhythmias, asthma	51226
10	61	F	82	129/79	Amiodarone, Moracizine, metoprolol	Paroxysmal palpitation	8470

BP = blood pressure; bpm = beats per minute; F = female; HR = heart rate; M = male; Pt = Patient; PVC = premature ventricular contraction.

### Experimental design

**Fig 1:**
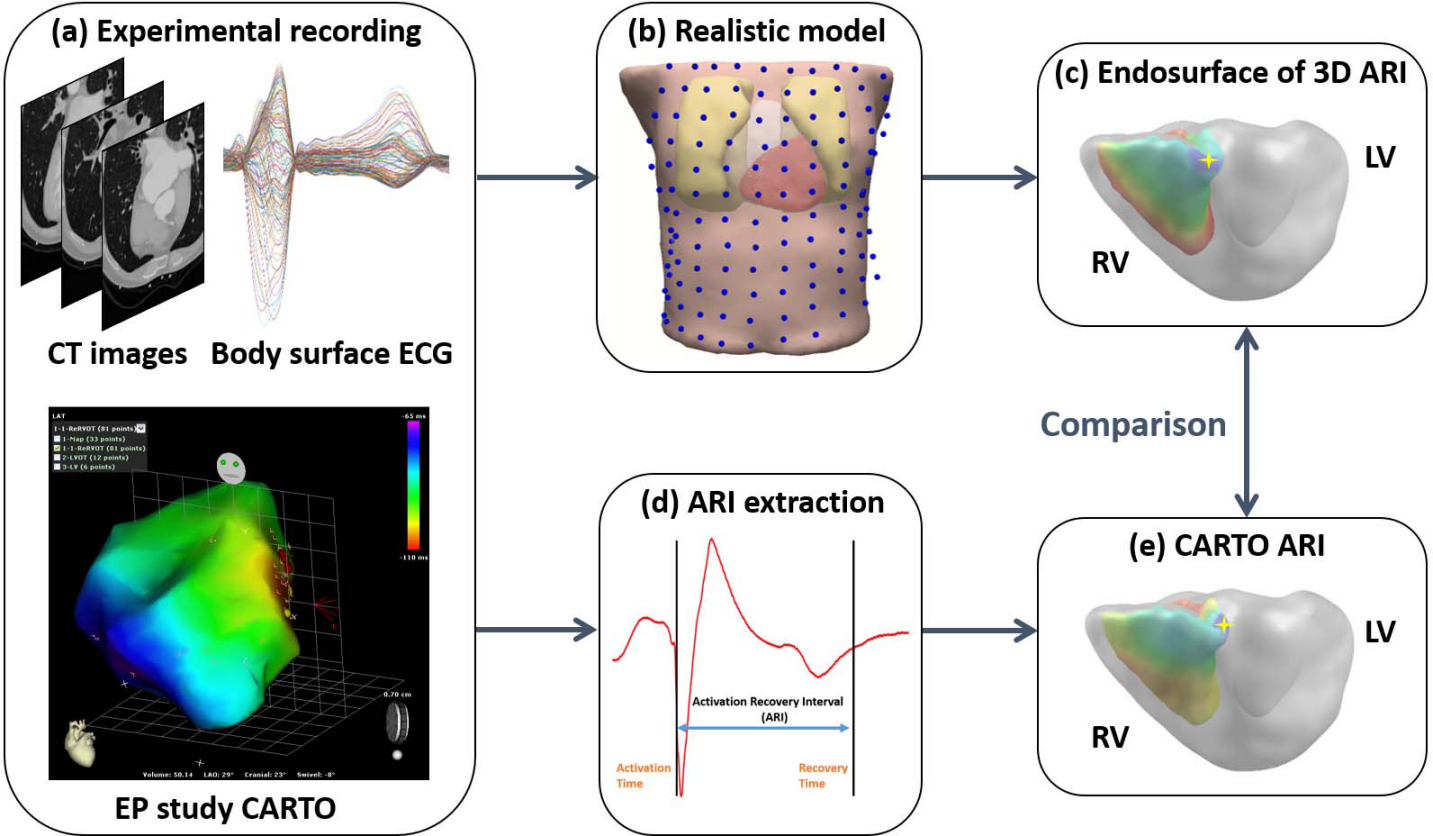
Schematic diagram of ARI imaging and validation in PVC patients. (a): Experimental recordings include cross-sectional CT slices showing detailed heart geometry and torso boundaries, butterfly plot of multiple-channel ECGs and local activation time maps recorded during EP study. (b): Realistic heart-torso model (blue dots showing body surface electrodes). (c): Endocardial surface of ARI maps by applying ARI imaging. (d): ARI extraction of individual recording sites from unipolar electrograms. (e): Interpolated endocardial surface of ARI maps from CARTO system. 3D = 3-dimensional; ARI = activation recovery interval; CT = computed tomographic; ECG = electrocardiogram; EP = electrophysiology; LV = left ventricle; PVC = premature ventricular contraction; RV = right ventricle.

Schematic diagram of the study is shown in **Fig 1**. CT images were collected before EP study, separately from body surface recording. An ECG-gated (70% R-R interval) contrast-enhanced cardiac axial CT scan from the level of great vessel to the diaphragm was performed to obtain the heart geometry with a resolution of 0.39*0.39*0.75 mm. Another thoracic scan from the collar bone level to lower abdomen with a resolution of 0.78*0.78*5 mm was to get a complete torso geometry. These two sets of CT images were registered based on important cardiac anatomical landmarks, such as the apex and the septum between two ventricles. The registration errors were minimized with the assistance of Curry 6.0. A realistic model including torso, lungs, epicardium, and ventricles was built from CT images by using a commercial software (Curry 6.0, Neuroscan, North Carolina, USA) for segmentation and registration.

Body surface recording was performed one day before EP study. 208 Ag-AgCl carbon electrodes were placed on the front (n = 140) and back (n = 64) of patients when they were in Fowler’s position. The electrodes were digitized by using an electromagnetic digitizer (Fastrak, Polhemus Inc., Colchester, VT, USA). We also digitized locations of anatomical landmarks on the body surface to aid co-registration of body surface electrodes with the torso CT images. These landmarks include acromion process, jugular notch, xiphoid process and umbilicus. The electrodes were then connected to BioSemi ActiveTwo system (BioSemi, Amsterdam, Netherlands) with a sampling frequency of 2k Hz to record body surface ECGs. Recording lengths were summarized in **Table 2**for each patient. Patients were asked to keep still with slow respiration to reduce motion artifacts during recording.

**Table 2:** Modeling and correlation analysis details.

Pt	BSPM Recorded (minutes)	Body Surface Nodes	Body Surface Triangles	Mean Triangle Edge Length (mm)	Ventricle Volume Points	Endocardial Surface Points	CARTO Endocardial Surface Points	Correlation Analysis Points	Unipolar Recording Site (No.)	Average Registration Error between CARTO and CT (mm)
1	10	3445	6886	10.76	3904	408	1486	408	41	3.24
2	24	1869	3734	15.62	6533	1085	1573	1085	34	5.45
3	12	4698	9392	10.7	6398	1050	1378	1378	13	6.13
4	14	1061	2118	20.52	3552	883	3195	883	40	3.93
5	21	4565	9126	10.8	3649	682	909	909	18	5.37
6	13	1320	2636	20.54	7513	1231	864	864	52	5.58
7	14	2396	4788	15.74	5800	1105	754	754	82	5.91
8	21	2066	4128	15.73	6764	1263	631	631	23	6.8
9	24	1667	3330	15.7	3603	815	546	546	11	4.36
10	17	2034	4064	15.76	3946	808	1431	1431	22	4.55

AT = atrial tachycardia; ARI = activation recovery interval; Av = average; BSPM = body surface potential maps; CC = correlation coefficient; Dis-actiari = spatial distance between the earliest activated site and the longest ARI site in 3D ARI map; LE-map = localization error between the longest ARI sites in imaging ARI map and CARTO ARI map; Pt = patient; PVC = premature ventricular contraction; RBBB = right bundle branch block; RE = relative error; RVOT = right ventricular outflow tract; VT = ventricular tachycardia.

After each EP study, we collected CARTO files, which include endocardial surfaces of either or both ventricles, endocardial local activation time maps, ablation sites and unipolar electrograms. The unipolar electrograms were obtained site by site sequentially by CARTO system. The endocardial surfaces of ventricles were registered with the segmented heart from CT images using a four-degree-freedom (locations and scale) rigid body registration method [55]. The CARTO endocardial geometries were automatically aligned based on similarities in shape without rotation. The registration was performed by minimizing the spatial distances between CARTO surfaces and endocardial surfaces from CT images. The average spatial distance between CARTO surfaces and endocardial CT surfaces, namely the average registration error, of each patient is listed in **Table 2**.

After registering body surface electrodes with the realistic model (Curry 6.0), a boundary element model (BEM) comprising torso, lungs and heart was built, and the conductivities were set as 0.2 S/m, 0.05 S/m and 0.21 S/m respectively. Modeling details are summarized in **Table 2**. By implementing ARI imaging technique, a 3D ARI map was reconstructed. On the other hand, ARI was extracted from unipolar electrograms and was interpolated to get a CARTO ARI map. 3D ARI map was compared with the CARTO ARI map quantitatively. Detailed descriptions of ARI imaging technique and ARI extraction from unipolar electrograms are stated in the following sections.

### Forward modeling and imaging principle of ARI imaging

Based on bi-domain theory [56], the discrete cellular architecture can be represented by a macroscopic continuum model which consists of two domains: the intracellular and extracellular domain. These two domains are connected by transmembrane currents flowing in between across a theoretical membrane with no thickness. Assuming a quasi-static condition, the electrical field within the volume conductor is governed by:

$$\nabla \cdot [(G_{i}+G_{e})\nabla \phi_{e}]=\nabla \cdot (-G_{i}\nabla \phi_{m})$$ (1)

where *G*_*i*_ and *G*_*e*_ are intracellular and extracellular conductivity tensors respectively, *ϕ*_*e*_ is the extracellular potential and *ϕ*_*m*_ is the transmembrane potential. If we define equivalent current density to be ${\overset{\to }{j}}_{\mathrm{eq}}=-G_{i}\nabla \phi_{m}$, then the above equation can be rewritten as:

$$\nabla \cdot [(G_{i}+G_{e})\nabla \phi_{e}]=\nabla \cdot {\overset{\to }{j}}_{\mathrm{eq}}$$ (2)

Thus, ${\overset{\to }{j}}_{\mathrm{eq}}$ can be the equivalent cardiac sources for generating cardiac electrical activities. To model the distributed current sources, the whole ventricular myocardium is discretized into *N* grid points. An orthogonal triple dipole is placed at each grid point to represent local vector field with an arbitrary direction.

According to the analytical derivation [57], at any time, the electrical potential at a given point on the torso surface is a linear superposition of all the potential fields generated by cardiac sources at that time. By applying BEM theory after the tessellation of relevant surfaces (torso, lungs and epicardium), this linear relationship is also reflected in the discrete matrix equation:

$$\phi_{b}(t)=LJ(t)$$ (3)

where *ϕ*_*b*_(*t*) is a *M×1* vector of body surface potentials at *M* electrode positions at time *t* and *J*(*t*) is a *3N×1* vector of the equivalent current density at *N* source locations at time *t*. *L* is an *M×3N* transfer matrix with the lead field between one electrode and one cardiac source is a *1×3* vector.

Since *3N* is usually much larger than *M*, finding *J*(*t*) given *ϕ*_*b*_(*t*) is an ill-posed inverse problem, which needs mathematical regularization. A generalized Tikhonov regularization is applied to solve this inverse problem:

$$\underset{J(t)}{\text{min}}({\left\|\phi_{b}(t)-LJ(t)\right\|}_{2}^{2}+\lambda {\left\|WJ(t)\right\|}_{2}^{2})$$ (4)

where *W* is the depth weighting matrix calculated from *L* and *λ* is the regularization parameter determined by L-curve method [58].

After solving the inverse problem on QRS complexes and T waves separately, the time course of the equivalent current density at each source location is obtained. By definition, the amplitude of equivalent current density is proportional to the spatial gradient of transmembrane potential. During the process of activation (or recovery), the spatial distribution of equivalent current density is dominated by its values at the interface between the activated and non-activated myocardium (or between the recovered and non-recovered myocardium). According to the peak criteria [34], the activation time *τ*_*a*_ (or recovery time *τ*_*r*_) is when the amplitude of *J*(*t*) reaches its maximum during the duration time *T* at a fixed location *P*:

$$\tau (p)=\underset{t\in T}{\text{max}}(J(p,t))$$ (5)

Activation recovery interval (ARI) is defined as the interval between recovery and activation, so ARI at location *P* is: *ARI*(*p*) = *τ*_*r*_(*p*)−*τ*_*a*_(*p*). Since ARI value is the difference between recovery time and activation time, it is a relative value. ARI imaging reconstructs the spatial pattern of ARI instead of obtaining the absolute value of ARI. Therefore, ARI imaging results were further linearly scaled based on the range of ARI estimated from BSPM. The longest possible ARI value corresponds to the length of a PVC beat and the shortest possible ARI value the sum of QRS length plus ST length.

### ARI extraction from unipolar electrograms

**Fig 2:**
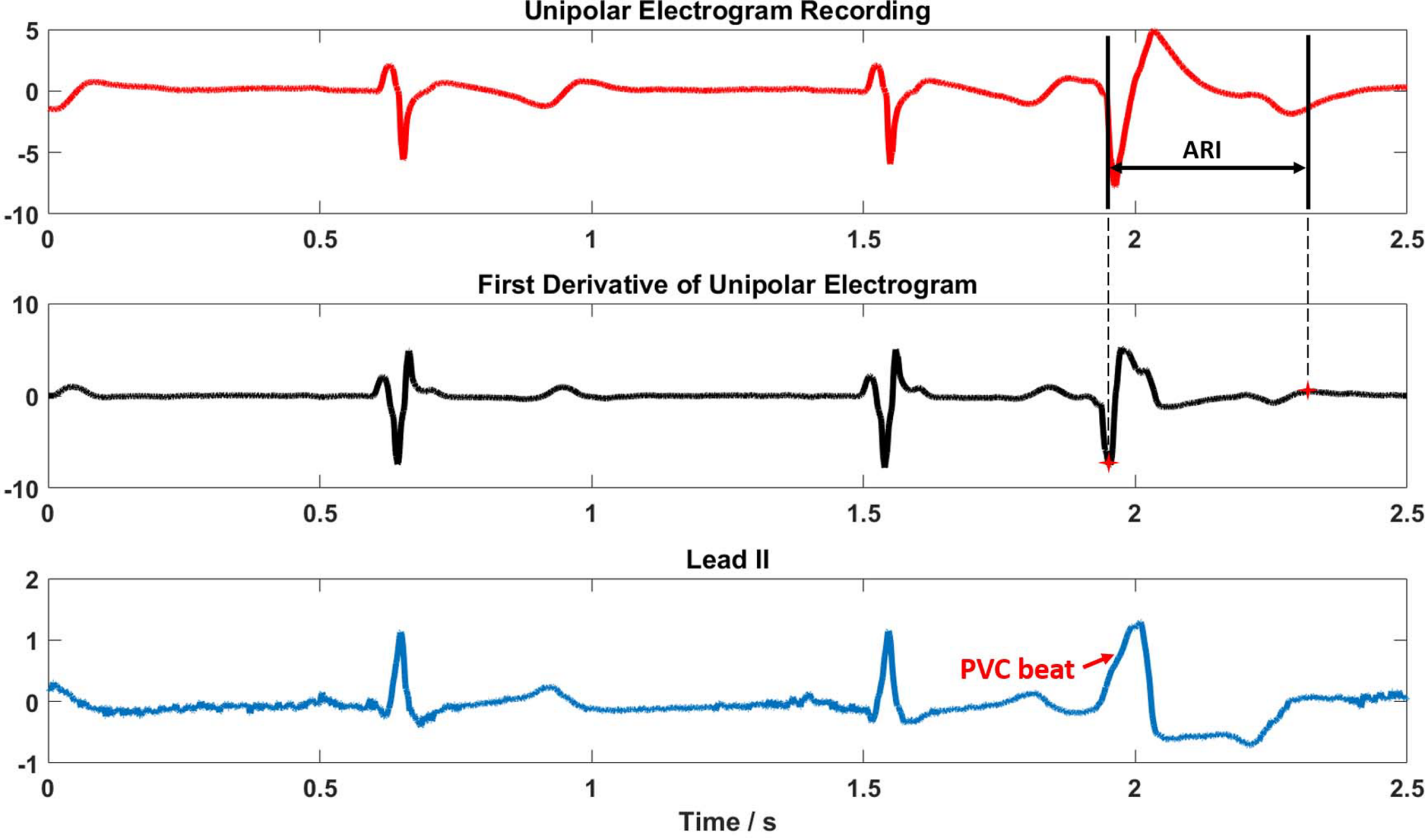
ARI extraction from unipolar electrograms. Top: Unipolar electrogram (M1) of a recording site. Middle: First derivative of the top panel. Bottom: Simultaneous recording of Lead II by CARTO system. Amplitude unit of recordings is mV, and time unit is second. ARI = activation recovery interval; PVC = premature ventricular contraction.

Unipolar electrograms were included in CARTO files. A quad mapping catheter was used. At each recording site, four unipolar electrograms (M1 to M4) were recorded along with 12-lead ECGs by CARTO system. The CARTO technician in the EP lab used 12-lead ECG to identify the PVC beats. We plotted M1 signal and the first derivative of this signal along with Lead II from 12-lead ECGs to help identify PVC beats in **Fig 2.** The time length of CARTO recording is 2.5 seconds in all the channels. The minimum derivative in QRS and the maximum derivative in T wave were automatically detected by a custom algorithm written in MATLAB and marked as activation time and recovery time respectively. In detail, the first derivative of the unipolar electrogram (M1 channel) was calculated. Then within the time segment of QRS complexes (or T wave), the minimum derivative (or maximum derivative) was automatically determined. The time window of calculating the first derivative is 20 samples with a sampling frequency of 1 kHz which means the first derivative at time point *i* is the difference between the magnitude at time point *i*+10 and *i*-10. Window length of 20 samples is to reduce the influence of high frequency noise. Therefore, the accuracy of determining the minimum (or maximum) derivative is 20 ms. However, this accuracy does NOT affect the accuracy of determining ARI from unipolar electrograms because ARI is a difference between recovery time and activation time. What influence this auto-detection algorithm had on determining the time point with the minimum derivative was imposed on in the same way of determining the time point with the maximum derivative. The criteria of determining activation (or recovery) time from unipolar electrograms is backed up by the studies mentioned in Introduction section. The results of ARI extraction from CARTO-recorded unipolar electrograms were considered as the ground truth in this study. We don’t have recordings from individual myocardial cells to validate the unipolar electrograms because the protocol doesn’t allow us to do so. Therefore, one ARI value was obtained at one recording site. Finally, ARIs at multiple recording sites were interpolated to the CARTO endocardial surface to get an endocardial ARI map for each patient by an inverse distance weighted (IDW) interpolation method [59].

### Statistical analysis

To evaluate the performance of ARI imaging, a quantitative comparison was made between two endocardial ARI maps: one was extracted from the 3D ARI map and the other was interpolated CARTO ARI map. Each patient had one interpolated CARTO ARI map and ten 3D ARI maps reconstructed from ten PVC beats in BSPM for comparison. For Patient 1, 2 and 4, unipolar recording sites spread throughout the whole chamber of either left ventricle or right ventricle, which enables us to extract the endocardial ARI map from 3D ARI map and project the endocardial CARTO ARI map to the endocardial surface from CT images. Projection from Volume A to Volume B was done by finding the closest spatial point in Volume B of the point in Volume A. Therefore, comparison was made on the endocardial surface from CT images in Patient 1, 2 and 4. However, for the other 7 patients, because of the small number of unipolar recording sites and the accompanying small region of CARTO endocardial map, 3D ARI map was projected to and CARTO ARIs were interpolated to the same CARTO endocardial surface for comparison respectively. Statistics include correlation coefficient (CC) and relative error (RE) between two endocardial ARI maps, localization error between the longest ARI sites (centers of mass with longest ARIs) in two maps (LE-map), and spatial distance between the earliest activated site and the longest ARI site in 3D ARI map (Dis-actiari). The standard deviation of CC is the standard deviation among ten obtained CCs, not of the distribution of any individual CC. CC, RE, LE-map and Dis-actiari were calculated as following:

$$CC=\frac{\sum_{i}\left(IARI_{i}-\bar{IARI})(MARI_{i}-\bar{MARI}\right)}{\sqrt{\sum_{i}{\left(IARI_{i}-\bar{IARI}\right)}^{2}}\sqrt{\sum_{i}{\left(MARI_{i}-\bar{MARI}\right)}^{2}}}$$ (6)

$$RE=\sqrt{\frac{\sum_{i}{\left(IARI_{i}-MARI_{i}\right)}^{2}}{\sum_{i}MARI_{i}^{2}}}$$ (7)

$$LE-map=\left\|S_{I}-S_{M}\right\|$$ (8)

$$Dis-actiari=\left\|S_{acti}-S_{ari}\right\|$$ (9)

where *IARI*_*i*_ and *MARI*_*i*_ represent the imaged and measured ARI at *i*_*th*_ location, $\bar{IARI}$ and $\bar{MARI}$ are the average values of the two sequences; *S*_*I*_ and *S*_*M*_ are the spatial locations of imaged and measured longest ARI sites respectively, *S*_*acti*_ is the earliest activated site in activation time map, and *S*_*ari*_ is the longest ARI site in 3D ARI map. CC, RE, LE-map, and Dis-actiari are included in **Table 3**.

**Table 3:** Statistics of ablation location and data analysis.

Pt	Diagnosis	Ablation Location	Recorded Longest ARI site	Imaged Longest ARI site	PVC beat (ms)	QRS (ms)	ST (ms)	T-wave (ms)	CC	RE	LE-map (mm)	Dis-actiari (mm)
1	PVC	RVOT free wall	RVOT free wall	RVOT free wall	362	101	130	131	0.83±0.01	0.03±0.01	4.1±0.6	4.1±0.9
2	PVC, RBBB	Left *Anterior* Fascicle	Left *Posterior* Fascicle	Left *Posterior* Fascicle	379	88	125	166	0.86±0.04	0.05±0.01	7.7±2.0	N/A
3	PVC, Nonsustained VT	RVOT free wall	RVOT free wall	RVOT free wall	362	113	78	171	0.84±0.02	0.04±0.02	3.4±2.3	4.1±2.5
4	PVC, Nonsustained AT	RVOT free wall	RVOT free wall	RVOT free wall	416	113	136	167	0.81±0.02	0.08±0.02	3.4±1.1	5.0±0.5
5	PVC	RVOT free wall	RVOT free wall	RVOT free wall	387	119	106	162	0.85±0.01	0.11±0.01	2.6±0.1	3.7±0.2
6	PVC, Nonsustained VT	RVOT free wall	RVOT free wall	RVOT free wall	394	126	89	179	0.84±0.01	0.08±0.01	5.9±1.0	4.8±0.8
7	PVC	RVOT free wall	RVOT free wall	RVOT free wall	411	111	144	156	0.94±0.01	0.06±0.01	3.6±0.3	3.2±1.0
8	PVC	RVOT septum	RVOT septum	RVOT septum	326	113	58	155	0.91±0.04	0.05±0.01	5.1±1.7	3.1±1.1
9	PVC	RVOT free wall	RVOT free wall	RVOT free wall	356	92	110	154	0.81±0.06	0.01±0.01	1.3±0.1	4.8±0.7
10	PVC	RVOT septum	RVOT septum	RVOT septum	350	97	84	169	0.86±0.03	0.06±0.04	7.1±2.0	3.5±1.3
**Av**	N/A	N/A	N/A	N/A	374	107	106	161	0.86±0.05	0.06±0.03	4.5±2.3	4.0±1.3

## Results and discussions

We have investigated, for the first time to our knowledge, the 3D ARI imaging to determine the spatial pattern of activation and recovery and to reconstruct ARI maps in PVC patients noninvasively. Quantitative comparisons are made between imaging results and clinical recordings. Prior to this study, ventricular recovery patterns could only be reflected from epicardial potentials [60] noninvasively or from endocardial potentials [61] invasively. Although activation pattern may remain unchanged, recovery could vary and thus plays an important role in the susceptibility to cardiac arrhythmias. Given the inhomogeneous and dynamic nature of recovery pattern, a 3D ARI imaging can provide important information guiding determination of the underlying arrhythmogenesis. We have shown the capability and performance of 3DCEI by imaging activation patterns under various conditions in the past. This study expanded our study scope to recovery in humans.

### Quantitative comparison and localization of the longest ARI site

A total of 100 PVC beats (10 for each patient) were analyzed. Each patient had 10 3D ARI maps calculated from 10 PVC beats in BSPM, which were compared with the patient’s one CARTO ARI map to evaluate the performance of ARI imaging. A good correlation was found with an average correlation coefficient (CC) of 0.86±0.05 and an average relative error (RE) of 0.06±0.03. The average localization error between the longest ARI sites in two maps (LE-map) is 4.5±2.3 mm. Based on the activation time maps of activation imaging results, PVC originated from right ventricular outflow tract (RVOT) in 9 out of 10 patients, 7 in the right ventricle free wall side and 2 in the septum side. One patient (patient 2) who was diagnosed as having right bundle branch block (RBBB) had the ectopic beats initiated from left anterior fascicle in the left ventricle. These are in accordance with the clinical ablation locations. From 3D ARI maps of ARI imaging results, the site with the longest ARI was at the same location as the clinical ablation location, with only one exception that for the patient with RBBB, the longest ARI site lied in left posterior fascicle in the left ventricle. Detailed statistics of data analysis were included in **Table 3**.

**Fig 3:**
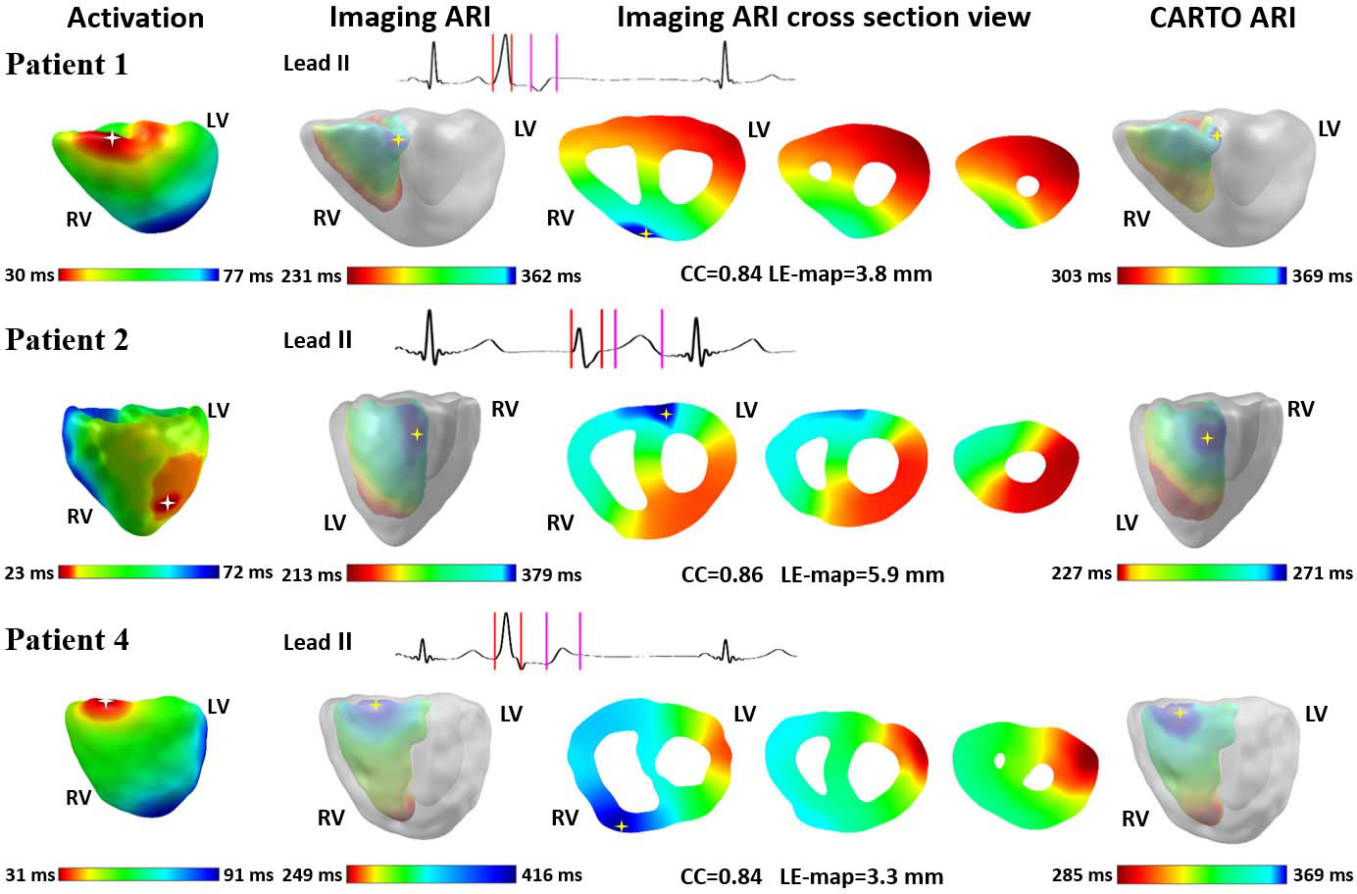
Results with recording sites covering a full chamber. **First column:** Imaged activation time maps. **Second column:** Endocardial surface of 3D ARI maps. **Third column:** Three cross section views of 3D ARI maps. **Fourth column:** Interpolated CARTO ARI maps. Yellow stars in the ARI maps represent the longest ARI sites. All the maps were color-coded from red to blue. 3D = 3-dimensional; ARI = activation recovery interval; CC = correlation coefficient; LE = localization error; LV = left ventricle; RV = right ventricle.

**Fig 3**shows the results for three patients (Patient 1, 2 and 4) with recording sites covering the full chamber. The first column are the imaged activation time maps, in which white stars represent the earliest activated sites. The second column refers to the imaged endocardial ARI maps and the fourth column the interpolated CARTO ARI maps. In both columns, the longest ARI sites are labeled by yellow stars. In between, there are three cross section views of imaged 3D ARI maps. All the maps are colored from red to blue, with red representing the minimum and blue the maximum. The time scales of imaging ARI maps are scaled based on the body surface recordings and the time scales of CARTO ARI maps are determined by the ARI values of recorded sites. The PVC beats analyzed were marked in Lead II electrograms. The time segment between red vertical lines was used for activation imaging and the time segment between magenta vertical lines was the input for recovery imaging. In patients 1 and 4, the earliest site in activation and the longest ARI sites in both ARI maps are in the same region, namely RVOT right ventricle free wall side. For patient 2, activation started at left anterior fascicle while the longest ARI site located at left posterior fascicle. The CC and LE-map are shown underneath.

**Fig 4:**
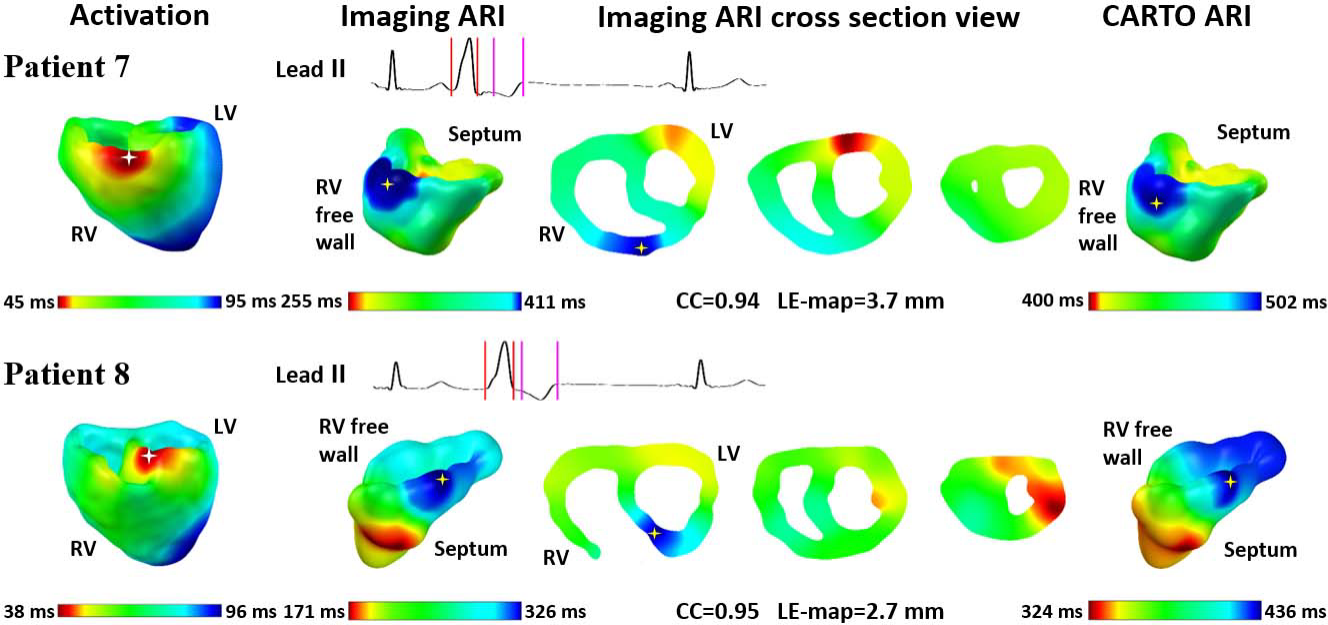
Results with fewer recording sites covering regional ventricles. The format is the same as in Fig 1. 3D = 3-dimensional; ARI = activation recovery interval; CC = correlation coefficient; LE = localization error; LV = left ventricle; max = maximum; min = minimum; RV = right ventricle.

Among the other 7 patients with small CARTO-recorded endocardial surfaces, 5 have the PVC beats starting from RVOT right ventricle free wall side and 2 are from RVOT septum side. Representative results of two patients are shown in **Fig 4**. Because of the small recording area, imaged ARI maps were projected to CARTO-recorded endocardial surfaces and then were compared with interpolated ARI maps quantitatively. The figure format is the same as that in **Fig 3**.

**Fig 5:**
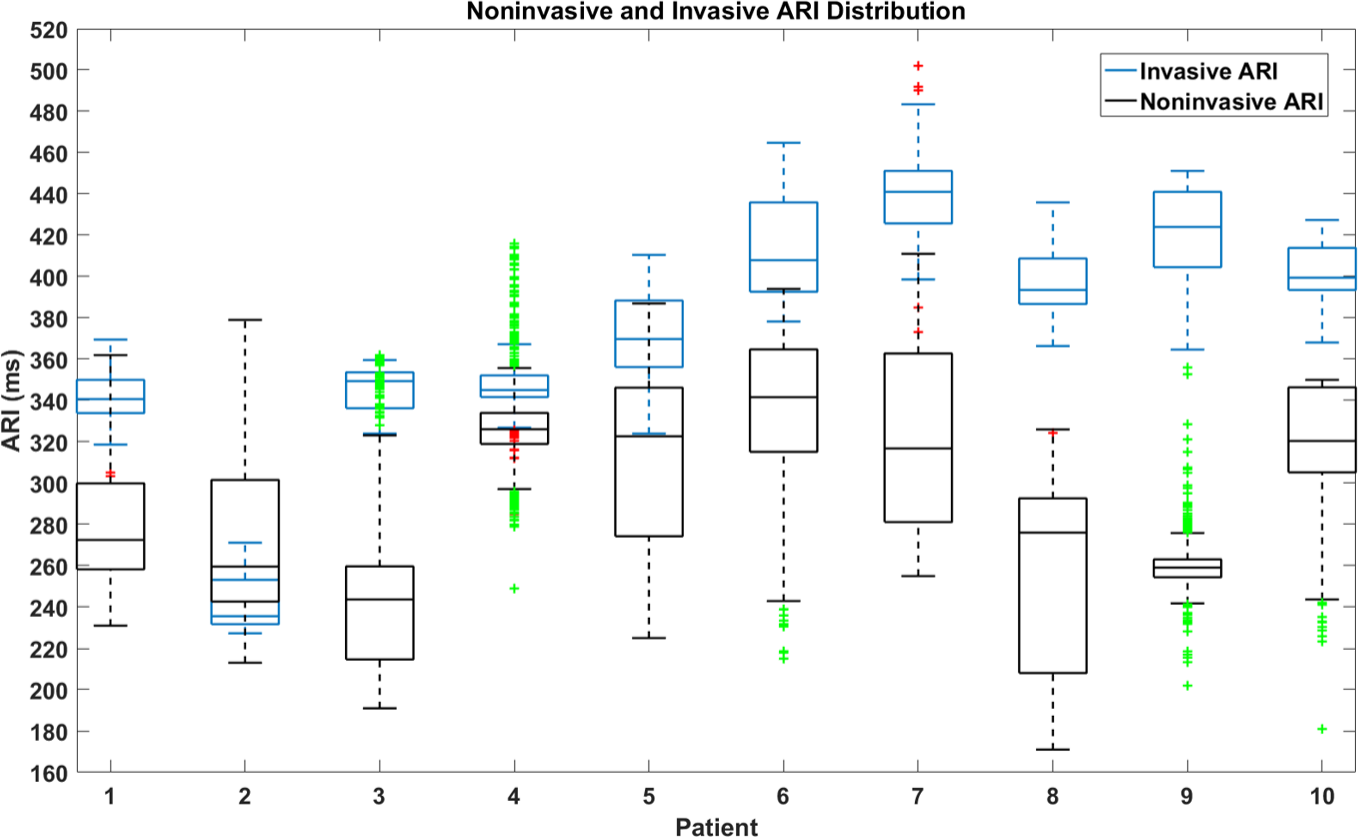
Extracted ARI distribution from CARTO recorded unipolar electrograms (blue) and Imaged ARI distribution (black).

**Fig 5**shows the standard boxplots of invasive CARTO ARI distribution from unipolar electrograms and noninvasive imaging ARI distribution in ten patients. Invasive ARI distributions are shown in blue boxplots and noninvasive ARI distributions are plotted in black boxplots. The median is the middle line in the box. The first quartile (q_1_) and the third quartile (q_3_) are the bottom line and top line of the box. Outliers are defined as values greater than q_3_+w× q_3_-q_1_) or less than q_1_-w× q_3_-q_1_), and w equals 1.5. The outliers of invasive and noninvasive ARI distributions are marked as red and green plus signs, respectively. In both boxplots, the number of data points is the same as the number of data points in correlation analysis in **Table 2**. Except patients in **Fig 3**, the CARTO ARIs were from recording sites around the target ablation area in the left seven patients.

**Fig 6:**
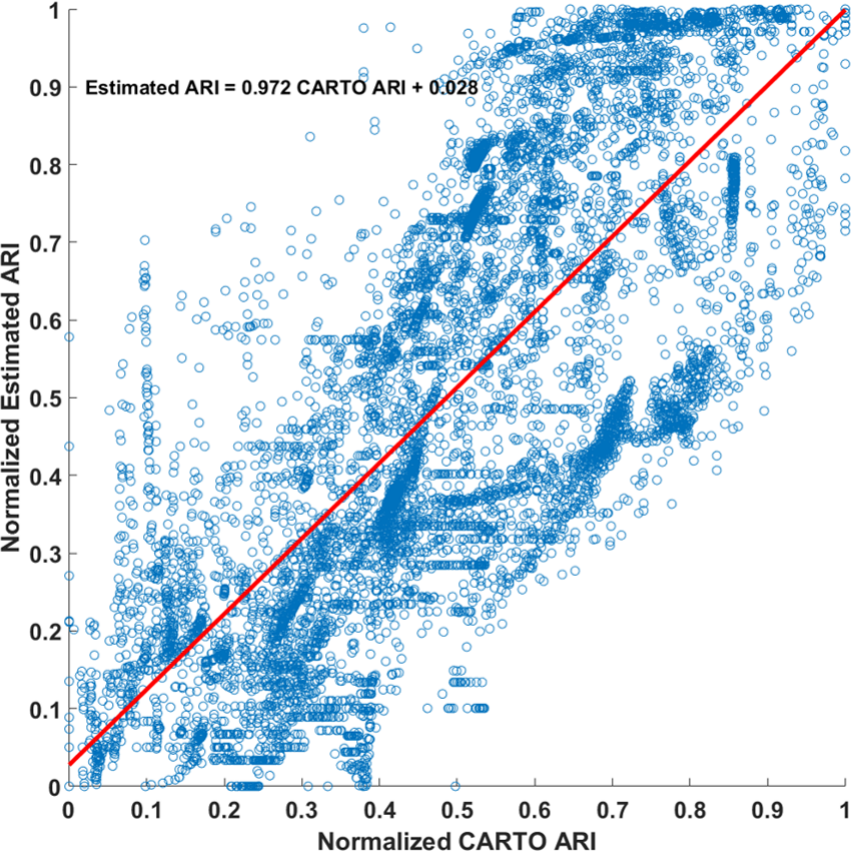
Scatter plot between normalized CARTO ARI and estimated ARI. Linear regression parameters are shown for the fitted line plotted.

**Fig 6**shows the scatter plot between invasive CARTO ARI and noninvasive imaging ARI. The values are normalized between 0 and 1 in order to plot for all patients. Linear regression was done between the two ARI data sets. Readers could check supplementary S1 Fig for the individual scatter plot of each patient.

In the previous noninvasive imaging studies [17,19,27], recovery patterns were shown, discussed and quantitatively compared. In the current study, we evaluated the performance of 3D ARI imaging by comparing the imaging results with ARI maps extracted from clinical unipolar electrograms quantitatively. Besides the three patients with recorded sites covering a full chamber, the other seven patients were mapped in a small region around the target ablation sites. So, the recorded ARI distribution in these patients covered the higher end of the ARI distribution from both ventricles.

### Clinical implications

During ectopic activities such as in PVC or pacing, recovery time is mainly determined by the activation sequence since activation time differences are much larger than local recovery (action potential duration) [60]. This is supported by previous findings in which activation time was inversely correlated with ARI during premature stimulation in patients with normal ventricles [61]. Our results are in accordance with the theory and previous findings in the aspect of detecting the longest ARI sites are close to the origins of PVCs in 9 out of 10 PVC patients with normal ventricles.

Discrepancy occurred in patient 2 who had RBBB in the ventricles. Normally, activation is conducted through both bundle branches of the conduction system. However, during RBBB, with the right bundle branch blocked, the right ventricle is activated by the impulses travelled through myocardium from the left ventricle instead of being activated through the bundle of His-Purkinje fibers and the left ventricle is still normally activated by the left bundle branch. Anatomically, the left bundle branch divides into the left anterior fascicles and the left posterior fascicles [62]. From the activation time map and the recorded successful ablation site’s location, PVCs were initiated by the left anterior fascicles in patient 2. What is interesting is that the longest ARI site was in the left posterior fascicles in both clinical ARI map and imaging ARI map. Whether this site would be the future potential origin of PVCs remains unclear and requires a follow-up observation. ARI imaging results of Patient 2 show that in patients with abnormal heart structure, the statement that the longest ARI site is close to the earliest activated site may not be true. For now, 3D ARI imaging has been reflecting ARI patterns faithfully in ectopic activities.

### Study limitations

**Fig 7:**
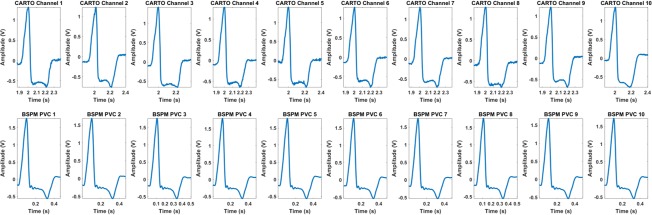
Plots of CARTO-recorded Lead II of PVC beats from ten endocardial recording sites and ten analyzed PVC beats on body surface Lead II. PVC = Premature Ventricular Contraction.

The patient population (n = 10) is small and the diversity of patients is limited since most patients have origins of PVCs at RVOT. PVCs are amongst the most homogeneous and gradual forms of recovery patterns. We did not investigate localized recovery gradients in this study. Seven patients were not mapped covering the full chamber, which may contribute to the higher correlation coefficients in some patients because of the projection and interpolation over a relatively small area. The limited number of recording sites and small regions of CARTO endocardial maps are due to clinical practice by the physicians. The current study design is not a simultaneous study between body surface potential mapping and CARTO recordings. CARTO recordings are sequential over multiple beats. However, these patients all had one type of monomorphic PVCs that was similar on 12-lead ECG as judged by the physicians. In **Fig 7**, we show that PVC beats from CARTO-recorded Lead II (top row) shared similar morphologies among different endocardial recording sites and these PVC beats were also similar in shape with the analyzed ten PVC beats recorded on body surface Lead II (bottom row). Ten endocardial recording sites throughout the right ventricle of Patient 1 were selected and displayed in the top row of **Fig 7**. Such similarity was also observed in all the other patients. Within these limitations, 3D ARI imaging promises to provide an insight into the global pattern of recovery and the capability of ARI in ectopic activities.

### Future application

3D ARI imaging can be potentially applied to any condition that involves recovery abnormalities in the ventricles. The current validation study is a first step towards validation of recovery imaging in other more complicated cases. This may include but not limited to: bundle branch block, early repolarization, cardiac memory, Wolff-Parkinson-White syndrome and ischemia. In the application of these cases, local recovery gradient will be added into the analysis. Also, to reflect the dynamic changes of recovery and to validate 3D ARI imaging, a simultaneous recording of BSPM and clinical recordings (contact or noncontact unipolar electrograms) should be performed.

## Conclusions

In this study, we have performed, for the first time to our knowledge, 3D ARI imaging of the recovery process in the ventricles. The good correlation and relative low localization errors as compared with clinical ARI maps suggest that ARI imaging could be an alternative of evaluating global dispersion of ventricular recovery in EP studies. The promising results suggest the 3D ARI imaging warrants further investigation to test the full potential of clinical applicability in a large number of patients suffering from various ventricular disorders.

## Supporting information

S1 Fig Scatter plots for individual subjects between CARTO ARI and estimated ARI. (TIF)
