# Supplementary figures and images for: Activation recovery interval imaging of premature ventricular contraction
Source: PLoS One. 2018 Jun 15;13(6):e0196916. doi: 10.1371/journal.pone.0196916 (PMC6003683; doi:10.1371/journal.pone.0196916)

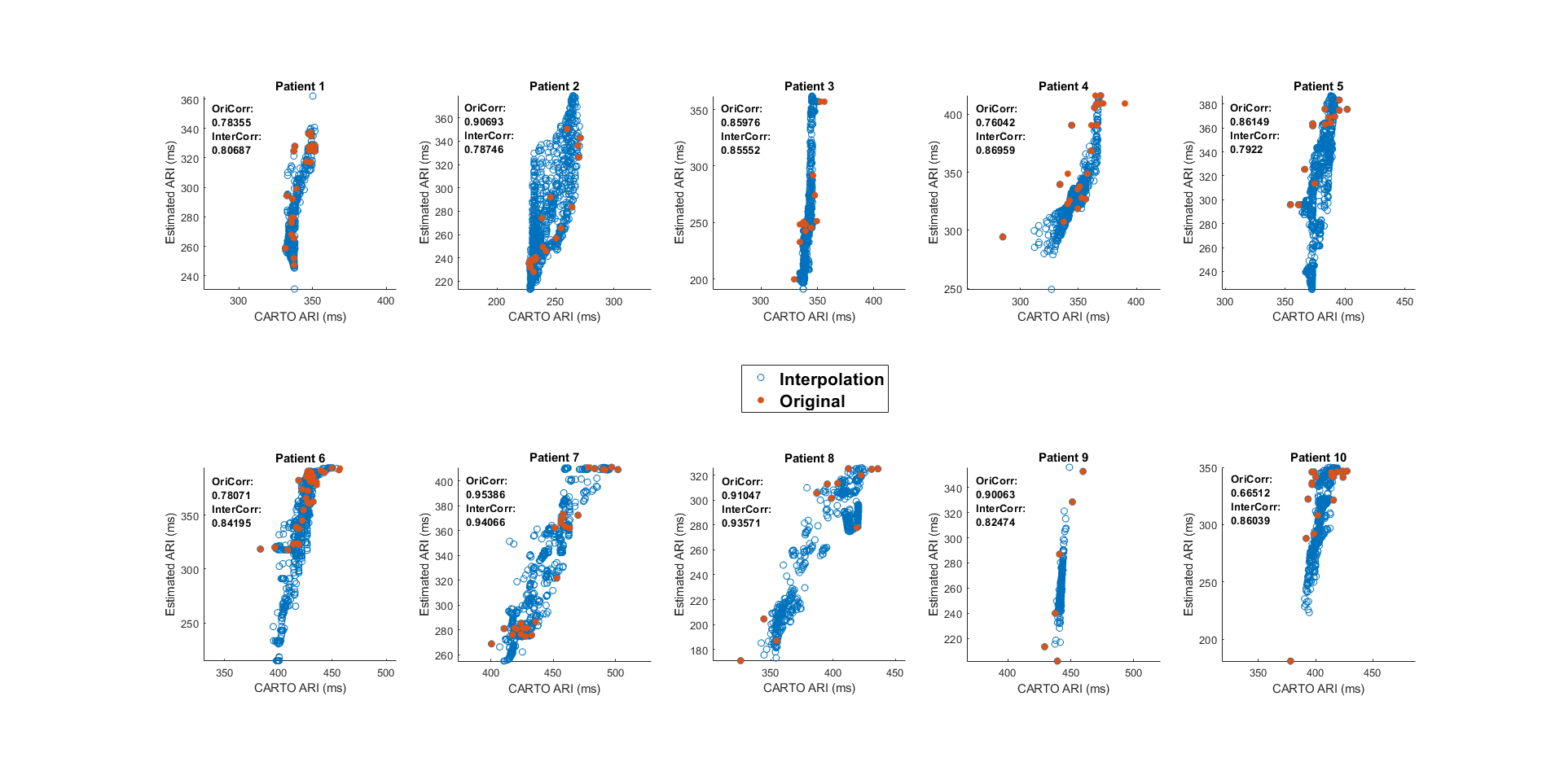

Supplement: S1 Fig — (TIF) [file pone.0196916.s001.tif]
